# Challenges to the implementation and adoption of physical distancing measures against COVID-19 by internally displaced people in Mali: a qualitative study

**DOI:** 10.1186/s13031-021-00425-x

**Published:** 2021-12-04

**Authors:** Mohamed Ali Ag Ahmed, Birama Apho Ly, Niélé Hawa Diarra, Fatoumata Bintou Traore, Djeneba Diarra, Inna Fatoumata Kande, Mahamadou Dembele, Seydou Doumbia, Hassane Alami

**Affiliations:** 1 Institute of Tropical Medicine, Antwerp, Belgium; 2 Faculty of Medicine and Odontostomalogy, University of Sciences, Techniques and Technologies of Bamako, Bamako, Mali; 3 Faculty of Pharmacy, University of Sciences, Techniques and Technologies of Bamako, Bamako, Mali; 4 Studies and Research Department, National Institute of Public Health (NIPH), Bamako, Mali; 5 Telfer School of Management, University of Ottawa, Ottawa, ON Canada; 6 University of Montreal, Montreal, Canada

**Keywords:** COVID-19, Coronavirus, Physical distancing, Displaced people, Internally displaced people, Mali, Sub-Saharan Africa

## Abstract

**Background:**

For almost a decade now, Mali has been facing a security crisis that led to the displacement of thousands of people within the country. Since March 2020, a health crisis linked to the COVID-19 pandemic also surfaced. To overcome this health crisis, the government implemented some physical distancing measures but their adoption proved difficult, particularly among internally displaced people (IDPs). The objective of this study is to identify the challenges relating to the implementation and adoption of physical distancing measures and to determine the main mitigation measures taken by IDPs to adjust to these new policies.

**Methods:**

An exploratory qualitative research was conducted in Bamako and Ségou, two of the ten regions of Mali. The study counted 68 participants including 50 IDPs, seven administrative and health authorities, and 11 humanitarian actors. Sampling was guided by the principle of saturation and diversification, and data was collected through semi-structured individual interviews (n = 36) and focus groups (n = eight). Analysis was based on thematic content analysis through NVivo software.

**Results:**

The main challenges identified concerning the implementation and adoption of physical distancing measures include the proximity in which IDPs live, their beliefs and values, the lack of toilets and safe water on sites, IDPs habits and economic situation, humanitarian actors’ lack of financial resources and authority, and social pressure from religious leaders. Implemented mitigation measures include the building of new shelters or their compartmentalization, the creation of income-generating activities and food banks, psychosocial support, promoting awareness of IDPs, and nightly police patrols and surveillance to discourage IDPs from going out. Finally, a call for action is suggested for the actors involved in IDPs support and management.

**Conclusions:**

The study demonstrates the difficulty for IDPs to follow most of the physical distancing measures and informs about the risk of disease spreading among IDPs with its potential consequences. It also shows the inability of mitigation measures to control the outbreak and suggests actions to be considered.

## Background

Internally displaced persons (IDPs) are generally overlooked in global health discussions [1]. Their status is more precarious and they benefit from even weaker protection than that of refugees [2]. They are also more vulnerable than the general population because they are poorer and more easily affected by health problems [3, 4]. COVID-19 has exacerbated their vulnerability and IDPs are often at higher risk of contact due to their inability to comply with public health measures [4]. Indeed, IDPs live in precarious living conditions, in overcrowded areas without basic amenities such as soap, running water, and medical services (4, 5). Over one-fourth of IDPs reside in low- and middle-income countries.

In Mali, the political and security crisis of 2012, pushed thousands of people to move to safer areas of the country [6]. According to the United Nations High Commissioner for Refugees, the number of IDPs is increasing in the country and was estimated to be 346,864 in January 2021 [7]. In general, these people are forced to leave their homes and economical activities to live in slums and are compelled to rely on small jobs or the solidarity of relatives, friends, political authorities, and humanitarian actors. Most of the time, they live in precarious conditions that expose them to health problems with limited access to health services.

On top of this political and security crisis, Mali has also been facing, since March 2020, a health crisis due to the COVID-19 pandemic. This pandemic quickly spread with 12,467 confirmed cases and 419 deaths as of April 14, 2021 [8]. In response to the crisis, Malian decision-makers authorities have introduced multiple public health measures in hopes of protecting populations from the pandemic. For centuries, these measures have been the cornerstone against epidemics [9]. They include physical distancing measures which aim to reduce interactions between potentially infected but not yet identified and thus not isolated individuals and other members of a community [10]. In Mali, these measures were introduced relatively quickly and were progressively applied using a top-down approach [11]. To ensure their rapid implementation, decisions were made most of the time by a national defense committee led by the President of Mali. Measures included the prohibition of gatherings of more than 50 people, including collective prayers, the closure of schools and universities, the suspension of commercial flights, the closure of borders, the implementation of a curfew, changes in work hours, the promotion of teleworking, and changes in workspaces [11]. These measures were considered by Malian decision-makers and authorities as most suitable in the local context. However, their effectiveness depends on their implementation and adoption by the local population [12]. Initial observations revealed multiple challenges regarding their implementation and adoption, particularly among IDPs whose living conditions are more conducive to the spread of the virus [13]. Additionally, most IDPs seem to have a low level of literacy and limited knowledge of the symptoms and mode of transmission of COVID-19 which further increases their risks. Many of these displaced people are not aware of the importance of basic preventive measures, notably physical distancing measures [14]. They sometimes seem to neither understand nor be aware of the recommendations given by authorities and are seldom convinced of their relevance. COVID-19 thus exposes them to a new threat that could end up being more dangerous than the events that led them to leave their lands and homes in the first place [15].

This study aimed to contribute to an effort to contextualize and adapt physical distancing measures targeting COVID-19 among IDPs in Mali. Specifically, it aimed to understand the challenges to the implementation and adoption of these measures among IDPs and the main mitigation measures put in place against the problem.

## Methods

### Design, and setting of the study

This study is an exploratory qualitative research conducted in Bamako and Ségou, two of the ten regions of Mali. These two regions were chosen for their security and accessibility compared to other regions. They are characterized by a high population density and a fast demographic growth. Around their regional capital, numerous spontaneous slums were created and assembled the most vulnerable families including IDPs whose access to health services is limited despite their high risk for infectious diseases.

### Study population and sampling

Participants were selected based on their knowledge and experience about the subject of our study. The study population includes (1) administrative and health decision-makers; (2) humanitarian actors involved in the management of IDPs, and (3) IDPs to whom physical distancing measures were applied. The list of categories of administrative and health decision-makers and humanitarian actors was identified in consultation with the head of the social development department in charge of coordinating the management of IDPs. Participants within these categories were selected using purposive sampling, and IDPs using snowball sampling. The selection was based on the principle of saturation and diversification [16, 17]. The sample size for saturation, according to Mason, averaged 31 participants [18]. Other researchers recommend 30 to 60 participants [19], while many recommend a minimum of 15 participants for qualitative research [20]. For the current study, 68 participants were included to ensure the completeness of the information received (Table 1). They were aged 18 years and over, without any cognitive troubles or COVID-19 symptoms, and were not in isolation or quarantine during the period of data collection. They were identified and selected with the support of the national and regional divisions of social development services.

**Table 1:** Distribution and number of participants to the study

	Bamako		Ségou		Total
	II	GI	II	GI	
IDPs	10	16	8	16	50
Humanitarian actors	5		6		11
Health and administrative authorities	4		3		7
Total	19	16	17	16	68

II: individual interview, GI: group interview

### Data collection

Data collection was conducted between November and December 2020 by two research teams, each including one research professional and two interviewers. It was facilitated by the national and regional divisions of social development services. Face-to-face semi-structured and individual interviews were conducted with IDPs from four IDPs sites in Bamako (Mabilé, Senou, Niamana, and Faladié) and two in Ségou (Zogofina and Bougounina). For health and administrative authorities and humanitarian actors, face-to-face individual interviews were performed according to their preference at their office or home. All interviews were recorded. Afterward, supplementary data was collected in March 2021 among four IDPs and two humanitarian actors to collect more information.

### Data analysis

Content analysis was chosen as the preferred method of data analysis. It allows for the interpretation of written text from interviews, observations, or documents [21]. More specifically, we used thematic content analysis, which is one of the most common and effective techniques [22]. It "consists of systematically identifying, grouping, and secondarily examining the discursive themes of a corpus" [22]. The analysis began with data collection by fully transcribing the data from the various audio recordings made. Transcription was validated by the research professionals (HD and NBT) and main researchers (MAAA and BAL). The resulting verbatim transcripts were then imported into the NVivo software to be analyzed inductively. Then, transcripts were examined line by line and paragraph by paragraph to generate codes labeled under units of varied sizes. Next, we grouped similar codes under one sub-theme, then under one theme. This process was independently carried out by two research professionals and a second screening was done by two principal researchers. Following this process, a thematic tree was created after a hierarchization of the themes according to their main or peripheral role in answering our questions. Their recurrence allowed us to have a synthetic representation of the content to analyze with different figure cases related to our study.

## Results

### Sociodemographic characteristics of participants

The mean age of participants was 35.37 years, with a minimum of 18 years and a maximum of 61 years. The most represented age range was 18 to 40 years. IDPs represented 73.5% of participants, the male gender dominated in Bamako and Ségou (41 men and 27 women). None of the participants has been tested positive for COVID-19 (Table 2).

**Table 2:** Description of participants according to sociodemographic data

Characteristics	Number of participants		
	Bamako	Ségou	Total
*Age range*			
18–40	26 (70.27)	11 (29.72)	37
41–60	18 (62.06)	11 (37.93)	29
61 and above	2	0	2
*Gender*			
Male	27 (65.9)	14 (34.1)	41
Female	19 (70.4)	8 (29.6)	27
*Type of participants*			
IDPs	37 (74)	13 (26)	50
Humanitarian actors	5 (45.5)	6 (54.5)	11
Health and administrative authorities	4 (57.1)	3 (42.9)	7
*COVID-19 history*			
Positive case	0	0	0
Suspected case	6 (75)	2 (25)	8
Contact case	4 (80)	1 (20)	5
Total			68 (100)

### Main challenges encountered in the implementation and adoption of physical distancing measures by IDPs

The main challenges recorded by participants are shown in Table 3.

**Table 3:** Summary of main challenges to the implementation and adoption of physical distancing measures

*Main challenges*
Proximity of households in IDPs sites
Values and beliefs on IDPs sites
Scarcity of water points and toilets in IDPs sites
IDPs habits, and lack of jobs and savings
Lack of financial resources and authority to enforce measures
Social pressure from religious leaders

#### Proximity of households in IDPs sites

This challenge was identified by a large majority of participants as a major one when implementing and adopting physical distancing measures. In general, IDPs live in small shelters which were found to be occasionally very overcrowded. This situation, according to some participants, rendered difficult the implementation and the adoption of physical distancing measures within households. The following quote illustrates this: *« …there are many people who are also part of large families, so it is very difficult to implement physical distancing. Imagine a family in a hut of four square meters for five to six people. How can physical distancing be applied in this context? (Male IDP_Bamako).*

Overcrowding in shelters was also found to be associated with the closing of schools that kept children at home and increased pressure on families. *"With schools closed, children are staying at home." (Male, IDP,_Bamako), " Ah! the difficulty is that children are at home with nothing to do…." (Male, IDP,_Bamako).*

Most notably though, IDPs simply prefer to stay close to each other to create solidarity and collectively face their problems. This strategy, according to participants, is the one used in their villages to face their problems.

#### Values and beliefs on IDPs sites

Participants indicated several values and beliefs which they considered to be very important for IDPs and which could be a barrier to the adoption and implementation of physical distancing measures. These values and beliefs include the need to participate in social events such as baptisms, marriages, funerals; to gather for lunch or dinner; to greet by shaking hands or hugging, and to visit people suffering from disease or other problems. *"We prefer to eat together using one plate. It reinforces our bonds and besides, we do not have enough plates to separate everyone’s food…"(Male IDP,_Ségou).* For participants, not respecting these values and beliefs could lead to stigma, discrimination, and social exclusion. *"It is badly seen if you do not participate in some social events. You’ll face stigma because it is not a good thing. You have to maintain solidarity." (Male IDP, Ségou).*

On top of these values and beliefs, some participants indicated that they do not believe in COVID-19 because they had never met someone who tested positive. *"What makes it harder is that people do not understand the disease because we hear about it, but we have never seen it. Really, we talk of coronavirus, but none of us have seen a sick person. That is how people think." (Male IDP_Ségou).*

#### Scarcity of water points and toilets in IDPs sites

Basic amenities are difficult to come by on IDPs sites. Participants confirmed that the scarcity of the water points in IDPs sites was a real challenge regarding the implementation and adoption of physical distancing measures. They mentioned that it pushes IDPs to line up for hours in front of public fountains just to obtain drinking water. *"Water is a problem because our supply here is very limited. Here there is challenging access to water. Displaced people have no water. Presently, we have a real water issue. Just look at the lines." (Female, IDP, Ségou).*

Additionally, the study revealed that most of the toilets in IDPs sites were public and that each of them was shared by several households. To access these toilets, IDPs sometimes must line up similarly to the water points. *"It is a bit complicated. You’ll see three families, sometimes five, who use the same toilet. I don’t see how we can apply physical distancing." (Male IDP_Bamako).*

#### IDPs habits, and lack of jobs and savings

Participants mentioned that IDPs are not used to staying home inactive. They are used to going out, either to look for work or other personal reasons. *"We are not used to staying home with nothing to do. Back in the village, we all had daily activities. It is the first time we are in such a situation." (Female, IDP,_Bamako).*

The lack of activities in their new places disables IDPs from earning money. This situation, compounded with their old habits, compels them to go out to look for jobs to feed their family, build a shelter, and create space for their family. *"In the morning, you have no resource for providing food while eating is a necessity. If you’re hungry, you need to go out to try and find something. When you have the means to eat, you can stay home." (Male IDP,_Ségou).*

These activities were found to be unsuitable for the adoption of physical distancing measures. According to participants, the lack of jobs on IDPs sites was augmented by the closing of borders and the curfew.

The problem is further exacerbated because IDPs lifestyle is characterized by living one day at a time and most of the participants disclosed that they do not have savings. Consequently, they must go out every day to earn money to fulfill their essential needs. This situation, according to participants, impeded the adoption of physical distancing measures. *"When you have no means, you can’t stay home." (Male, IDP,Bamako), "We can try our best to avoid getting the disease, but not leaving our homes when we have nothing, that’s hard." (Male, IDP, Ségou).*

#### Lack of financial resources and authority to enforce measures

IDPs revealed that it is difficult to stay home and respect measures without support from the government and humanitarian actors. They are expected to adopt the measures without support for hunger.

*"If the poor have no help it’s difficult. If you stay home to protect yourself from the disease, well you’ll get another illness and it’s called hunger. That’s all I have to say." (Male IDP,_Ségou).*

This poor support system was explained by the insufficiency of financial resources of humanitarian actors and decision-makers. They have noted limited resources that do not allow them to properly assist IDPs in respecting physical distancing measures. These resources are notably insufficient to provide for IDPs daily needs or to build decent shelters with enough space for maintaining physical distance. *"Despite our willingness to help displaced people respect measures, at a certain level our resources as a humanitarian organization are insufficient to take care of everything." (Male, humanitarian actor, Bamako).*

Decision-makers and humanitarian actors further expressed their inability to make some physical distancing measures respected, particularly the closing of religious spaces, the curfew, and the prohibition of gatherings for funerals, marriages, or baptisms. *"In general, I do not have the required authority to make all prevention measures respected…." (Male, health decision-maker, Bamako), "We do not have the authority to impose the measures." (Male, health decision-maker, Bamako).*

#### Social pressure from religious leaders

Participants also noted ambiguity resulting from decision-makers not clearly ordering the closing of places of worship due to pressure from religious leaders while forbidding gathering for collective prayers. Additionally, some IDPs maintain that their religion demands that they pray as groups at the mosque and that physical distancing is thus difficult to respect.

*« In the mosque, it is not easy to physically distance because even if you were trying to, someone could come and stand right next to you and you can’t just chase them, especially when there is no more room in the mosque. Then, we are also told that in a mosque, it is good to stand next to each other when praying, that’s when our prayers are better received by God*» *(Male IDP, Bamako).*

### Main mitigation measures to facilitate the implementation and adoption of physical distancing by IDPs

To face the above-mentioned challenges, decision-makers, humanitarian actors, and IDPs implemented several local mitigation measures as shown in the Table 4.

**Table 4:** Main mitigation measures to facilitate the implementation and adoption of physical distancing measures

Building of new shelters and compartmentalization of existing ones to separate family members,
Financial aid with the creation of income-generating activities (IGAs) and food banks,
Raising awareness of COVID-19 among IDPs through diverse communication tools,
Nightly police patrols and surveillance by humanitarian actors protection agents to dissuade IDPs and limit their mobility

### Building of new shelters and compartmentalization of existing ones to separate family members

Although their means are minimal, IDPs created other shelters to provide more space for their families*: "When we make a little money, we build extra huts to use as stores or for part of the family. This allows us to gain a little more space” (Female, IDP, Bamako).*

Other IDPs compartmentalized their shelters in two or three areas to separate family members*: « We divided shelters in two rooms, an antechamber, and a veranda. Adults stay in the room and children under the veranda. If you have the resources, you can build another shelter in the courtyard». (*Male, IDP, Ségou).

### Financial aid with the creation of IGAs and food banks

Additionally, the government and humanitarian actors provided financial aid, some income-generating activities (IGAs), and food banks to mitigate the impact of physical distancing measures and facilitate their adoption by IDPs.

*« To fulfill the needs of families greatly affected by the pandemic, income-generating activities were implemented. The government and its partners must find ways to help them revive their activities. Instead of catching a fish for someone every day, you must instead teach them to fish. That’s empowerment». (*Male, IDP, Ségou).

### Raising awareness of IDPs through diverse communication tools

Efforts were also made to raise awareness of IDPs through the media and television sketches. They were particularly made aware that physical distancing measures apply to all, including IDPs.

*“We later realized that many IDPs do not speak Bambara, which is the most widely spoken language in Bamako. We varied the outreach techniques in their language to reach them, which seemed to yield better results. We also used translated materials to communicate with them about prevention measures against COVID-19»* (Male, humanitarian actor, Bamako).

### Nightly police patrols and surveillance by humanitarian actors protection agents to dissuade IDPs and limit their mobility

Dissuading measures such as nightly patrols by police and humanitarian protection agents were put in place to dissuade IDPs from leaving their homes.

*« We do both, raising awareness and threatening. Some protection agents pretend to take down the information of those who go out at night, all to dissuade them from doing so. That’s it» (*Male, humanitarian actor, Bamako).

These mitigation measures were deemed efficient by participants, although some believe they ought to be reinforced.

## Discussion

This study provides additional scientific evidence highlighting the vulnerability of IDPs to epidemics. It confirms what other studies have shown, that compared to host communities, IDPs are generally more likely to contract diseases such as COVID-19 because of their precarious living conditions related to the loss of their means of subsistence and income [3, 4, 23, 24]. These authors highlighted how COVID-19 exacerbated several social inequalities between populations within the same country. Although the public health measures implemented to prevent COVID-19 were difficult for the entire population to comply with, they were easier to implement in the general population than among IDPs because of their specific constraints [5]. This study contributes to the understanding of these constraints and proposes some actions to mitigate them. Thus, it fills an important knowledge gap regarding the neglected and isolated population of IDPs [25] notably in sub-Saharan Africa. The qualitative approach used was more appropriate to address the research questions as the subject is poorly documented. Main identified challenges relate to all physical distancing measures introduced in Mali, particularly the prohibition of mass gathering, the curfew, the closure of schools, universities, and the borders. These challenges are mainly linked to IDPs’ living conditions in proximity, limited financial resources, the failure of authority and leadership to ensure compliance to the preventive measures, the beliefs and the strong attachment of IDPs to their social values, and the lack of safe drinking water and toilets at home which forces IDPs to scramble around these services. It could be safely said that these challenges are similar to those encountered by numerous populations living in precarious conditions, notably in slums, and who are estimated to count for a billion people worldwide [26]. However, these challenges seem to be more difficult for IDPs in Mali because of their great vulnerability. Similarly, other studies have found that in highly populated displaced camps without safe drinking water, compliance to social distancing measures is difficult [15, 27, 28]. Indeed, the structure of these camps because of insufficient space does not allow for the respect of social distancing measures [4, 29]. Additionally, displaced peoples’ shelters are situated so close to each other that a few steps outside of a shelter find you in a neighbor’s land. Displaced people often use silk curtains or plastic screens to create the illusion of living in separate rooms. Furthermore, the number of toilets and water fountains in these camps is insufficient, which leads displaced people to use the same spaces [30, 31]. These living conditions not only make physical distancing impossible but also limit cross ventilation which is vital to reduce the potential of aerial transmission of COVID-19 [30]. They exacerbate existing social and health vulnerabilities which can lead to even more grave results related to COVID-19 among displaced people [27].

All participants agree that the insufficiency of financial resources requires outdoor activities to address the basic needs of IDPs and presents itself as a major challenge to the implementation and adoption of physical distancing measures. To meet their daily needs, IDPs are constrained to strong mobility that does not permit them to adopt these varied measures. The humanitarian and governmental actors who provide them with assistance neither have the means to guarantee them minimal vital needs nor can they offer them shelters that can improve the respect of physical distancing. Some researchers have similarly noted that generally, it is difficult to make public health measures respected by a poor population fighting for survival [31, 32].

Deeply established values and social habits were also highlighted as a major challenge to the adoption of some measures, namely the prohibition of gathering for marriages, funerals, or collective prayers. These events negatively affect the rapid spread of COVID-19 and their prohibition is essential to limit the disease [33]. IDPs participate in such activities to remain close to each other, discuss, and create solidarity to face their situation, which is not favorable to physical distancing.

The study also helped to identify the main implemented mitigation measures that proved to be relatively efficient but very insufficient. Consequently, a call to action is made for authorities and humanitarian actors to act in favor of IDPs.

### Call to action

The Malian crisis has been ongoing for close to a decade now, and the security situation only seems to deteriorate. Consequently, the number of IDPs living around the cities is increasing. Many observers agree that IDPs will have to remain in camps for a long time while the short-term hope of a return to regular life weakens in their villages of origin. As new COVID-19 waves are happening, it becomes urgent to implement immediate actions to protect very vulnerable IDPs. We should not wait for more damages to happen, as is often done, before acting. All actors involved in the management of IDPs should be acting towards identifying the needs of IDPs and meeting them to limit the spread of the pandemic. That is why we are making a call for action to help contain the spread of epidemics or pandemics such as COVID-19.

Among actions that need to be privileged, it is important to relocate IDPs to new sites with more space and greater distance between shelters which will favor physical distancing and create conditions for a better lifestyle. Doing so will involve planning spaces to build decent shelters with the appropriate supply of safe drinking water and familial toilets. In Mali, there is enough space in the cities’ periphery to install IDPs. What is needed is financial resources for which advocacy should take place among technical and financial partners for their contribution. Additionally, the relocation of these IDPs should be accompanied by the creation of income-generating activities and schools for children to decrease their dependency on the cities and their mobility.

Additionally, this study demonstrated that IDPs often do not understand Bambara or French which are spoken in Ségou and Bamako. This particularity explains their disconnect to mass awareness campaigns. It is important to make available educational material in their languages, adapt messages destined to them and the channels used to specifically answer their questions and concerns. These measures were highlighted as being important in other studies focused on finding sustainable solutions to fight COVID-19 [24, 34]. Some authors plead for co-creation and co-production of solutions with displaced people to create messages adapted to their reality [35].

These actions should be complemented with the prioritization of IDPs during COVID-19 vaccination campaigns to limit the spread of the disease. Similarly, improved access to health services to ensure isolation and support of COVID-19 positive cases outside of family homes seems urgent. Urgent also is the quarantining of contact cases outside of camps to protect their relatives.

### Strengths and limits of the study

This study is one of the firsts that tackle the issue of physical distancing among IDPs. It allowed us to meet IDPs in their camps and collect their very rich opinions thanks to thorough interviews. It was realized by professionals with in-depth knowledge of the environment. Identifying challenges regarding the implementation and adoption of physical distancing measures by IDPs seem to constitute a major contribution to existing knowledge which serves as a guide towards the COVID-19 response in this vulnerable environment. Therefore, the study informs authorities and humanitarian actors on the importance of improving the COVID-19 response among IDPs. Those are the strengths of the study.

Like all qualitative studies, these results cannot be generalized outside of the sites in the two regions where the study took place. However, collected data was remarkably similar to, and reflected the difficulties noticed by other researchers among displaced people around the world. The results could thus be transferable to other contexts with similar characteristics. Additionally, we realized during the analysis of the initial collected data, that some measures were less discussed by the participants. We returned on site to ensure completeness of the data and to verify its validity among a few participants. Transcriptions were also often crossed with our field notes and all divergences were discussed and clarified.

## Conclusions

This exploratory qualitative study helped demonstrate that implementation of the physical distancing measures targeting the transmission of COVID-19 is not feasible by IDPs. IDPs are confined in small spaces on overpopulated sites and limited resources make it difficult to create adequate conditions in respect of these measures. The study highlights the important risk of the disease spreading on IDPs sites with potentially dramatic consequences. Implemented mitigation measures seem to contribute to improving the situation of IDPs but are still insufficient. Finally, a call to action is made to mobilize actors involved in the management of IDPs, and a non-exhaustive list of actions was proposed to improve the adoption of physical distancing measures. It is important to prioritize displaced people who are increasing in number in Mali and around the world. Complementary studies will be valuable in testing and implementing interventions targeting the improvement of the adoption of physical distancing measures among IDPs.

## Data Availability

The datasets generated and/or analyzed over the current study are available from the corresponding author on reasonable request.

## Abbreviations

IDPs: Internally displaced people
IGA: Income generating activity
II: Individual interview
GI: Group interview
